# The Hospitals Association and Patients' Contributions

**Published:** 1888-07-07

**Authors:** D. Walsh, S. Benton


### The Hospitals Association and Patients' Contributions.

Speeches by Dr. Stoker, Dr. Jamison, Mr. D. Walsh, Surgeon-Major Ince, and Mr. S. Benton.

*(Continued from p. 210.)*

Dr. Stoker: I venture to offer a few observations with regard to the paper we have heard read, and also with regard to some of the criticisms which were expressed at the last meeting. We were told upon that occasion that this Pay System had more or less started in Guy's Hospital, where patients were charged a uniform sum. That is not the Pay System, which is a system which charges people according to their means. The other system is obviously wrong, because if you ask all the persons who come to you to pay a sum of fourpence, you are sure to get some who could only really afford twopence, and others who could really afford sixpence. There was one gentleman who devoted himself to anathematizing special hospitals. I do not hold a brief for special hospitals, but inasmuch as the strongest examples of the Pay System were drawn from special hospitals, it is fair to say a few words about them; and in the first place I should like to ask those gentlemen who help up general hospitals over special hospitals because the special hospitals only studied particular portions of the human anatomy, while the general hospitals studied the whole, whether, if they had something the matter with their eyes, they would go to the gentleman whose duty it is to vaccinate. Do the patients go to those special hospitals because they get better treatment than from the ordinary practitioner? No, it is because they get something for nothing—and that is a very wise reason too. Mr. Carr-Gomm said that if the patients paid small sums, they became too independent and difficult to keep within discipline. I do not hold with that, and I have had experience in hospitals, both with the Pay and the Non-pay system. At the London Hospital they have 20 men and 200,000 attendances per annum. It is impossible that those 20 men can inquire into the reasonableness of the claims of those 200,000 patients. The essence of the system is in this—that we want the responsibility of the character of the patients to rest with the hospital authorities, and then the patients would have to prove their wants. It is impossible for any charitable or medical institution to refuse patients the first time they come; but if any patient comes to the hospital and states that he is necessitous, he must be handed a voucher, and told that the next time he comes he must produce that, filled up. The voucher may be filled up by a respectable householder. One of the great objections of hospital authorities to the Pay System is the question of teaching; and then there are the questions of finance and practicability. Mr. Brudenell Carter stated at the Mansion House that we should pay the people for coming to the hospitals, because they played an all-important part in teaching the students. Now, at the London Hospital, the working days for clinical instruction are not more than 250. There are 800 patients per diem; and suppose that there are 400 students. If these students spent a quarter of an hour in examining each patient, they would be occupied from six in the morning until five at night in seeing half the number. Supposing there were ten of them on duty each day, and they spent five minutes on each case, it would take them six hours a-day. So much for the teaching side of the question. As to the financial side of the question, I do not think it is an exaggeration to say that 20 per cent. of the people who go to the London Hospital are able to pay something, and if that 20 per cent. put a sixpence each, there would be £1,000 a-year. Further than that, each out-patient costs the London Hospital 4*s.* a-year, and if 10 per cent. were wiped out, there would be a clear gain of another £1,000 a-year. Money saved is money gained, and I think that now I have pointed out a clear gain of £2,000 a-year. The instantaneous effect of the adoption of such a system would be, that people who had left the general practioner and gone to the general hospital, would, when they found that they had to pay something and wait a considerable time, return to the general practitioner. If it is only for this reason, we ought to try it. The system has been tried and found successful in some general and several special hospitals, and it is absolutely impossible for those who are opposed to it to try and thwart the onward progress of the Pay System—for it is irresistible.

Dr. Jamison: Allow me to state the experiences of a hospital in a large manufacturing town, which is so intensely dirty that the greater proportion of the people who work in it live out of it. This is the town of St. Helens, in Lancashire. The hospital is not endowed, and all the funds come from the artisan population. Every member of the artisan population in London coming to a London hospital would be thought poor, and would be relieved. Mr. Nixon spoke about coalheavers, but if he were to go to Lancashire and inquire into the Lancashire and Cheshire Relief Society, he would find that there are 25,000 members who pay for all accidents which occur in the mines. It is not compulsory, because, if it were, it would come under the Truck Act. It is purely voluntary, and there is a Cottage Hospital, every bed of which is free. Voluntary subscriptions amount to £260, and the men at the works subscribe over £800. It was put to the workmen that it was a great shame that they should "sponge," and that they ought to be ashamed of themselves to want other people to pay when they were sick. It was urged that they should put together sufficient funds to pay for themselves when they were taken ill; and a scheme was adopted by which, at nearly all the works, 1*d.* a-week was deducted by the clerk who paid the wages, and this sum was handed over to the hospital. It may be said that in London there would be a great difficulty in doing this, and the amount of hospital relief which is given free in London would represent 1*s.* 2*d.* a-week for every artizan. It seems to me that Londoners will combine in every possible way for strikes and for demonstrations of every kind, but will not combine to pay for hospitals. It is a great boon to the town to have the hospital in the way I have described, because all sicknesses and accidents are provided for. They have no provident dispensary, but they have a system of paying in one penny a-week for advice, and every man in the place, and every family, have the means of claiming medical advice for which they have paid. At one works, where four hundred men were employed, they paid the doctor themselves, and he got £200 a-year. If certain works in London paid at that rate, you would have more than the hospitals want. I hope I have put the plan clear, because I see no reason why what is possible in small towns should not be possible in large. In St. Helens they had so much money that they were able to send all their convalescent cases away to a convalescent hospital, for which they paid 10*s.* each patient a-week. The improvement has been equally great in regard to medical practitioners in the town, and the whole profession of the place was brought to an infinitely higher standard. Every one of the works had its own doctor attached, and the scheme works very satisfactorily for the doctors.

Mr. D. Walsh: The system of subscribers' letters, as one giving admission to hospitals, is a bad one, in my opinion. A poor person labouring under serious illness is frequently sent to get a letter, for which he sometimes makes the round of the neighbouring public-houses, generally with bad results.

These letters of admission lie at the root of a lot of mischief, for it puts a great deal of power into the hands of subscribers, however careless they may be. As to the relations between the medical profession and the hospital system, there is a very strong feeling amongst the rank and file that the Pay System should not be adopted in hospitals in any form whatever, and that a great deal of harm might be done by paying patients. It is desirable, in my opinion, to restrict and limit expenditure to the one great object—the relief of the sick poor.

Surgeon-Major INCE, while acknowledging with thanks the industry and care bestowed by Mr. Burdett-Coutts on investigating the subject, differed entirely from him as to the advisability of introducing the Pay System. He said: I am entirely opposed to the suggestions he makes in regard to imposing payment upon patients in hospitals. The principle of self-help is one of the highest importance, and one which should be most strongly encouraged; but I do not see why this principle should be brought forward as an excuse for asking for money for the sick poor. Why should they be subjected to payment? A suffering patient is to have his pain aggravated by knowing that an inquiry is being made into his private circumstances. Depend upon it, gentlemen, that when you try to enforce this system of making people pay, you will be utterly unable to calculate the moral harm and the mental distress which you will cause. I most strongly oppose the introduction of the Pay System. While I appreciate Mr. Burdett-Coutts' paper, and the boom which it will produce in the public mind, I must express my opinion that to apply the Pay System to the general hospitals is a great mistake, a cruel mistake. The system adopted in Lancashire is a different thing altogether, and there is as much difference between hospitals and provident dispensaries. In order to make up the deficit in hospital funds we ought to go to the clergy. I am sorry the Archbishop of Canterbury is not present to-night. Church funds is the source from which the hospital deficit should be made up. The clergy have appropriated funds which righteously belonged to the sick poor, and we should go to them to help us make up the deficit.

Mr. S. BENTON: My opinion is that those people who are in a position to pay towards their maintenance in hospital should pay, and that the onus of proving that they are unable to pay should rest upon themselves. Those people who pay are far more grateful than those who do not. The system as practised at St. Thomas's Hospital is one which I do not desire to see any of the great hospitals in London adopt, because of all systems of which I am acquainted it is the worst, for it injures a very worthy class of people—the general practitioner. The best example of the Pay System is the hospital in Fitzroy Square, which does no harm to the general practitioner, and which is conducted in such a way that everyone will admit it cannot be improved upon. The public are doubtless benefited by the system at St. Thomas's Hospital; and they get the very best nursing it is possible to have. My remarks are not directed against the staff, for I have received every courtesy from the resident medical officer of the institution; but I find fault with the system. A patient who is one day a patient in the hospital finds himself the next day a patient in the St. Thomas's Home. I do not think this is fair to the patients, or fair to the staff, for it is not their fault. It is not to be wondered at that gentlemen interested in general hospitals should find fault with special hospitals.

After some remarks from Mr. SAMUEL HILL,

The CHAIRMAN said: At this late hour I do not propose to detain you for any length of time. The paper is one of very great importance, and if we were to discuss it at all adequately we should each have to deliver a speech as long as Mr. Burdett-Coutts's paper. I should like to dwell upon one or two points, and say, in reference to the statements of Mr. Benton as to St. Thomas's Home, that many of the statements are entirely inaccurate. With regard to payment for treatment in hospitals, I feel a very great deal of sympathy with Mr. Burdett-Coutts's views, and yet I cannot say that I entirely agree with them. I agree with Sir Sydney Waterlow in regard to our large hospitals, and personally I should be sorry to see them accept pay. There is no doubt the charity of the public is not sufficient to maintain them, however, and it is only fair, where this is the case, that the funds should be in some way supplemented by the subscriptions of those who use them. The hospitals are really in debt, and require funds, and it is difficult to see how the matter is to be got over. The whole subject is a very perplexing one. One other point I wish to draw attention to is this, that we hear a good deal of talk about artisans, as if artisans alone were entitled to consideration. That seems to be to me entirely a mistake. There are servants, and governesses, and many other classes of people—people who work in shops, clerks, and people of that sort, who are in every way as deserving of the benefits of hospitals as the artisan classes. These are persons who do not and cannot provide for sickness, even in the way that the artisan classes can do. In conclusion, I desire to point to the very great importance of this subject, and to say that if it could be considered by a committee, say, of the governors and medical men of the large hospitals in London, who could carefully consider it and discuss it fully, and that under the auspices of The Hospitals Association some such meeting could be appointed which could go into the matter and consider Mr. Burdett-Coutts' paper thoroughly, it would be a very great thing. I hope that the ball which Mr. Burdett-Coutts has set rolling will have some good result.

A GENTLEMAN IN THE MEETING said: I wish to answer the gentleman who said he was ashamed of his profession for complaining of people coming into the hospital when they could afford to pay a medical man. I am not ashamed. These hospitals were intended and were originally built and started for the benefit of those who could not pay, and they were not started for the benefit of people who could pay. I do not think there are many general practitioners here. I will throw down this challenge, that if you will call a public meeting of general practitioners, and make it at four in the afternoon, when hard-working practitioners can attend, you will find that the feeling of all general practitioners is against this paying scheme. Since this paying system has been going on at Guy's, a large number of persons who would have been able to pay a small fee go to the hospital, and when you tell them they ought to be ashamed to receive charity, they tell you they are not receiving charity, but are paying for what they have. I ask you to call a meeting of general practitioners in a hall which would hold a good number, and you will find that what I say is true—that the whole profession is against what you propose.

